# Optimization of Extraction and Antioxidant Activities of Resveratrol from *Polygonum cuspidatum* by Ultrasound-Assisted Natural Deep Eutectic Solvent Method

**DOI:** 10.3390/molecules31030492

**Published:** 2026-01-30

**Authors:** Ying Guo, Siyi Wan, Yue Gu, Ting He, Zhaoyuan Chen, Xiaoxiao Qu, Jiaxin Quan, Junkai Ma, Izni Atikah Abd Hamid

**Affiliations:** 1 Shiyan Key Laboratory of Biological Resources and Eco-Environmental Protection, College of Chemistry and Environmental Engineering, Hanjiang Normal University, Shiyan 442000, China; guoying@hjnu.edu.cn (Y.G.); wansiyi@hjnu.edu.cn (S.W.); guyue@hjnu.edu.cn (Y.G.); heting@hjnu.edu.cn (T.H.); chenzhaoyuan@hjnu.edu.cn (Z.C.); quxiaoxiao@hjnu.edu.cn (X.Q.); 2 Hubei Key Laboratory of Wudang Local Chinese Medicine Research, Department of Chemistry, School of Pharmacy Hubei University of Medicine, Shiyan 442000, China; 3 Centre for Green Bioprocess Engineering, Faculty of Engineering, Built Environment and Information Technology, SEGi University, Jalan Teknologi, Kota Damansara, Petaling Jaya 47810, Selangor, Malaysia

**Keywords:** ultrasound-assisted extraction (UAE), natural deep eutectic solvent (NADES), resveratrol, *Polygonum cuspidatum*, antioxidant activity

## Abstract

*Polygonum cuspidatum*, a traditional medicinal plant widely cultivated in Hubei Province, China, contains resveratrol, which has been shown to regulate lipoprotein metabolism, inhibit platelet aggregation, and aid in the prevention of arteriosclerosis and cardiovascular diseases. However, conventional extraction methods are often limited by low efficiency and solvent toxicity. A novel extraction strategy integrating an ultrasound-assisted extraction with natural deep eutectic solvents (NADES) was developed to achieve environmentally friendly and effective recovery of resveratrol from *Polygonum cuspidatum*. The optimized NADES system consisted of betaine and DL-malic acid in a 1:4 molar ratio with 50% water content. Using single-factor experiments and Response Surface Methodology, the following parameters were identified as optimum: solid–liquid ratio, 1:28 g/mL; ultrasonic power, 240 W; ultrasonic temperature, 40 °C; and ultrasonic time, 30 min. In such a case, the resveratrol yield reached 33.12 mg/g by UV-Vis spectroscopy and 2.95 mg/g by HPLC analysis, significantly higher than that obtained by other methods. Antioxidant assays demonstrated that the extract exhibited strong scavenging activity against ABTS^+^•, DPPH•, and •OH radicals. These results demonstrate that the ultrasound-assisted extraction with NADES method provides an efficient and eco-friendly alternative for extracting resveratrol from *Polygonum cuspidatum*, yielding extracts with notable antioxidant properties.

## 1. Introduction

**Scheme 1:**
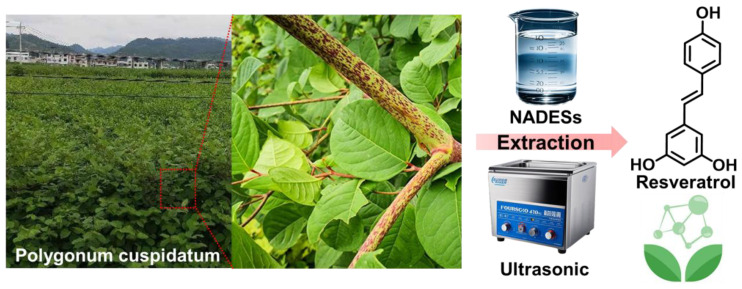
Ultrasonic-assisted extraction (UAE) of resveratrol from *P. cuspidatum* with natural deep eutectic solvents (NADES).

*Polygonum cuspidatum* (*P. cuspidatum*) is a large herbaceous perennial plant and a traditional and popular Chinese medicinal herb, officially recorded in the Chinese Pharmacopoeia [1]. This plant, extensively spread in southern China, has roots that are utilized as an effective agent [2]. *P. cuspidatum* has been used to treat arthralgia, chronic bronchitis, jaundice, hypertension, and hypercholesterolemia [3,4]. Resveratrol (3,5,4-trihydroxystilbene), a significant component of *P. cuspidatum* for pharmacological activities [5], is a stilbenoid polyphenol (Scheme 1) [6]. Resveratrol has physiological activities that have been shown to prevent cancer cells from migrating and spreading [7], in addition to having antioxidative, fatty liver-reducing, anti-obesity, anti-inflammatory, cardioprotective, neuroprotective, and anticancer qualities [8,9,10].

Previously, a variety of extraction methods were used to extract resveratrol from *P. cuspidatum*, like heating reflux [11], alkaline extraction [12], enzymatic hydrolysis [13], microwave-assisted extraction [14], supercritical CO_2_ extraction [15,16], and high-speed counter-current chromatography (HSCCC) [17,18]. However, several weaknesses were identified in these extraction processes, including complex conditions, low efficiency, prolonged duration, and high costs. Extraction efficiency can be improved with ultrasonic technology, which also reduces costs, shortens ultrasonic times, enhances product quality, and increases yield [19,20]. Choosing the right solvent is crucial for effective flavonoid extraction. When water was used, the extraction ratio and purity were low [21]. Researchers have used alkaline and organic solvents, such as sodium hydroxide aqueous solution, methanol, ethanol, and acetone [22,23,24,25,26,27], to improve extraction ratios. While these solvents increase efficiency, they can cause environmental pollution and raise production costs. Therefore, it is necessary to develop a green, highly efficient solvent for extracting resveratrol from *P. cuspidatum*.

In recent years, natural deep eutectic solvents (NADES) have found wide application as green extraction solvents, since they are considered a natural alternative to conventional organic solvents or ionic liquids [28]. NADES are systems formed by mixing natural hydrogen bond acceptors (HBA) and hydrogen bond donors (HBD). The HBA component is typically derived from anions, such as those from quaternary ammonium salts, or from the carboxylate groups of amino acids. In contrast, the HBD component commonly consists of hydroxyl- or carboxyl-containing compounds, including sugars, organic acids, and alcohols. These constituents, such as sugars, organic acids, and amino acids, are widely present in living organisms [29]. Compared with conventional organic solvents and ionic liquids, NADES offer significant economic and environmental advantages due to their inherent properties such as biodegradability, low toxicity, recyclability, and low cost [30]. Furthermore, they exhibit a range of excellent physicochemical properties, including the ability to remain liquid at sub-zero temperatures, tunable viscosity, a broad polarity range, and strong solubilizing capacity for diverse substances [31,32,33].

In this study, a series of NADES was designed and coupled with ultrasound-assisted extraction (UAE) for the extraction of resveratrol from *P. cuspidatum* (Scheme 1). The extraction conditions were optimized using a Central Composite Design (CCD) within Response Surface Methodology (RSM) to maximize yield [34,35,36]. Furthermore, the antioxidant activity of the optimized extracts was evaluated. This work establishes a feasible and environmentally friendly process for the efficient extraction of resveratrol.

## 2. Materials and Methods

### 2.1. Materials and Reagents

*P. cuspidatum* roots were procured from a local traditional Chinese medicine store (Shiyan, China). Plant material was powdered (180 μm) using a grinder (Model FW100, Yongkang, China) and then stored at room temperature before extraction.

Resveratrol, choline chloride (ChCl, >98%), citric acid (CA, >98%), betaine (Bet, >98%), L-lactic acid (L-Lac, >98%), glucose (Glu, >98%), DL-malic acid (DL-Mal, >98%), D-fructose (D-Fru, >98%), urea (>98%), glycerol (Gly, >98%), and ethylene glycol (EG, >98%) were purchased from Anhui Zesheng Technology Co., Ltd. (Hefei, China). The radical reagents 2,2-diphenyl-1-picrylhydrazyl (DPPH) and 2,2′-azino-bis(3-ethylbenzothiazoline-6-sulfonic acid) (ABTS) were supplied by Energy Chemical Co. (Shanghai, China). Distilled water was provided by Hangzhou Wahaha Co., Ltd. (Hangzhou, China). All other chemicals were of analytical grade.

### 2.2. Preparation of the Standard Curve of Resveratrol

**Figure 1:**
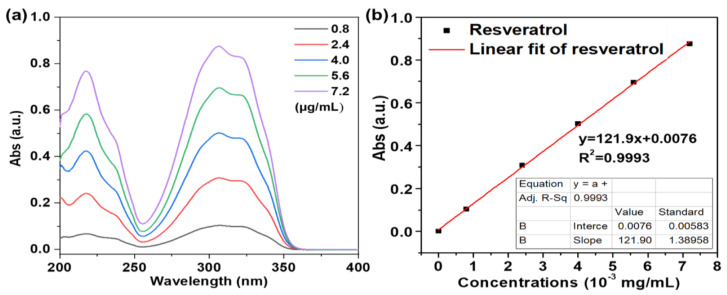
(**a**) Standard curve of resveratrol by the characterization of UV−Vis experiment; (**b**) linear fit of resveratrol concentration and UV−Vis absorbance.

A standard resveratrol solution (8 μg/mL) was prepared by dissolving 2.0 mg of resveratrol in ethanol, transferring and diluting it to a 250 mL volumetric flask. Aliquots (0, 1.00, 3.00, 5.00, 7.00, and 9.00 mL) of the above prepared standard solution were pipetted into a series of 10 mL volumetric flasks. Following measurement of their UV-Vis absorbance at 306 nm, a standard curve for resveratrol was generated by plotting absorbance versus concentration. Linear regression of the data yielded the equation y = 121.9x + 0.0076 (R^2^ = 0.9993), confirming a strong linear relationship between absorbance and concentration (Figure 1).

### 2.3. Ultrasound−Assisted Extraction Experiments

*P. cuspidatum* powder was mixed with the NADES in a conical flask for ultrasonic-assisted extraction (UAE). All samples were subjected to ultrasonication in a fixed position using an ultrasonic cleaning bath (SCQ−7201C, Shanghai, China). Based on a previous report with modifications [37], the extraction conditions were optimized as follows: a solid−liquid ratio of 1:25 (g/mL), an ultrasonic time of 30 min, an ultrasonic temperature of 40 °C, and an ultrasonic power of 240 W. The extract obtained was placed in a high-speed centrifuge (Model TDZ5−WS, Changsha, China) for 10 min. This supernatant was then sampled, diluted to 200 times, and stored in a dark place at 4 °C until further analysis.

The diluted extract was analyzed by UV−Vis spectrophotometry (Model 7200, Shanghai, China) to determine the absorbance of resveratrol at 306 nm. This concentration (μg/mL) was calculated based on the standard calibration curve established in Section 2.2.

### 2.4. Single−Factor Experiment

Since the UAE of NADES is employed to extract resveratrol from *P. cuspidatum* in this study, a single−factor experiment was designed and performed to evaluate the effect of extraction parameters on resveratrol yield. According to the extraction method, different extraction factors such as the solid−liquid ratio (1:15, 1:20, 1:25, 1:30, and 1:35 g/mL), ultrasonic power (200, 240, 280, 320, and 360 W), ultrasonic temperature (30, 40, 50, 60, and 70 °C), and ultrasonic time (10, 20, 30, 40, and 50 min) were investigated using single-factor experiments.

### 2.5. Statistical Analysis

For the Response Surface Methodology, analysis of variance (ANOVA) was performed using Design-Expert software (Version 8.0 X, Stat-Ease Inc., Minneapolis, MN, USA) to evaluate the significance of the regression model and each model term. The significance level was set at *p* < 0.05. The goodness of fit of the model was assessed by the coefficient of determination (R^2^), adjusted R^2^, predicted R^2^, and the lack-of-fit test. IC_50_ values for antioxidant activities were calculated by nonlinear regression using GraphPad Prism software (Version X, GraphPad Software, San Diego, CA, USA).

### 2.6. RSM-CCD Experiment

In order to further screen out factors that significantly affected the extraction yield, a Central Composite Design (CCD) of Response Surface Methodology (RSM) was adopted. In this experiment, four parameters were picked to further explore the optimum performance of resveratrol (Y) based on single−factor experiments. As shown in Table 2, factors and their ranges for RSM optimization were selected as follows: solid−liquid ratio (A) from 1:5 to 1:45, ultrasonic power (B) from 80 to 400 W, ultrasonic temperature (C) from 20 to 60 °C, and ultrasonic time (D) from 10 to 50 min. A total of 30 experimental runs were conducted, and the corresponding response values are presented in Table 3.

### 2.7. Preparation of P. cuspidatum Extract

Precisely 0.04 g of the powdered *P. cuspidatum* sample was weighed into a conical flask. The NADES composed of betaine and DL-malic acid at a molar ratio of 1:4 was prepared. Precisely 5 mL of this NADES was mixed with 5 mL of water (resulting in 50% water content). Then, 1 mL of this NADES mixture was accurately pipetted into the conical flask, establishing a solid−liquid ratio of 1:25 (g/mL). The mixture was homogenized and subjected to ultrasound−assisted extraction under the following conditions: ultrasonic power of 240 W, temperature of 40 °C, and extraction time of 30 min. The resulting extract was filtered through a 0.22 μm microporous membrane to obtain the resveratrol test solution, which was stored protected from light in a refrigerator prior to HPLC analysis. Analysis was performed using a ZORBAX SB−C18 column (250 mm × 4.6 mm, 5 μm). The HPLC conditions were as follows: the mobile phase consisted of methanol and water (40:60, *v*/*v*) at a flow rate of 1 mL·min^−1^, the injection volume was 10 μL, the detection wavelength was 306 nm, and the column temperature was maintained at 35 °C.

### 2.8. Antioxidant Activity Experiments

#### 2.8.1. ABTS^+^• Radical Scavenging Activity

The ABTS^+^• radical scavenging activity of the extracted resveratrol was evaluated according to a published protocol [38]. About 5 mL of K_2_S_2_O_8_ solution (2.45 mmol/L) and 5 mL of ABTS**^+^•** solution (7 mmol/L) were mixed for the reaction to occur for 12 h at 25 °C. At 734 nm, the absorbance of the mixture was adjusted to 0.7 ± 0.05 by adding distilled water. Subsequently, a volume of 400 μL of resveratrol extract solution (0.05, 0.10, 0.15, 0.20, and 0.25 mg/mL) was combined with 4 mL of the mixture solution in an incubator at 25 °C for 6 min. Then, the absorbance of the mixture solution was measured. The ABTS**^+^•** scavenging activity was determined using Equation (1):

(1) $${\mathrm{ABTS}}^{+}•\;\mathrm{scavenging}\;\mathrm{activity}\;\left(\%\right)=\left[1-\frac{A_{1}-A_{2}}{A_{0}}\right]\times 100\%$$

*A*_1_ is the absorbance of ABTS**^+^•** solution with the sample solution, *A*_0_ is the absorbance of ABTS**^+^•** solution without the sample solution, and *A*_2_ is the absorbance of the sample solution.

#### 2.8.2. DPPH• Radical Scavenging Activity

The DPPH• radical scavenging activity was measured according to a reported method, with slight modifications [39]. Briefly, 2 mL of the resveratrol extract solution at varying concentrations (0.10, 0.20, 0.30, 0.40, and 0.50 mg/mL) was mixed with 2 mL of a 0.2 mmol/L DPPH• solution. The mixture was incubated in the dark at 37 °C for 30 min, and the absorbance was then measured at 517 nm. A control was prepared by replacing the extract with an equal volume of solvent. The DPPH• scavenging activity was calculated using Equation (2):

(2) $$\mathrm{DPPH}•\;\mathrm{scavenging}\;\mathrm{activity}\;\left(\%\right)=\left[1-\frac{A_{1}-A_{2}}{A_{0}}\right]\times 100\%$$

*A*_1_ is the absorbance of the DPPH**•** solution with the sample solution, *A*_0_ is the absorbance of the DPPH**•** solution without the sample solution, and *A*_2_ is the absorbance of the sample solution.

#### 2.8.3. Hydroxyl Radical (•OH) Scavenging Activity

The •OH scavenging activity was determined as reported by Deng et al. (2021) [40]. First, 2 mL of resveratrol extract solution (0.05, 0.10, 0.15, 0.20, and 0.25 mg/mL), salicylic acid−ethanol solution (2 mL, 8 mmol/L), H_2_O_2_ solution (2 mL, 8.8 mmol/L), and FeSO_4_ solution (2 mL, 6 mmol/L) were successively transferred into a centrifuge tube at 37 °C for 30 min. At 510 nm, the absorbance was measured. The •OH scavenging activity was determined using Equation (3):

(3) $$•\mathrm{OH}\;\mathrm{scavenging}\;\mathrm{activity}\;\left(\%\right)=\left[1-\frac{A_{1}-A_{2}}{A_{0}}\right]\times 100\%$$

*A*_0_ is the absorbance of the control group. It is only the absorbance of salicylic acid and FeSO_4_. *A*_1_ is the absorbance of the sample solution, salicylic acid, H_2_O_2_, and FeSO_4_. *A*_2_ is the absorbance of the sample without the sample solution but adding H_2_O_2_.

## 3. Results and Discussion

### 3.1. Screening of the Optimal NADES

**Figure 2:**
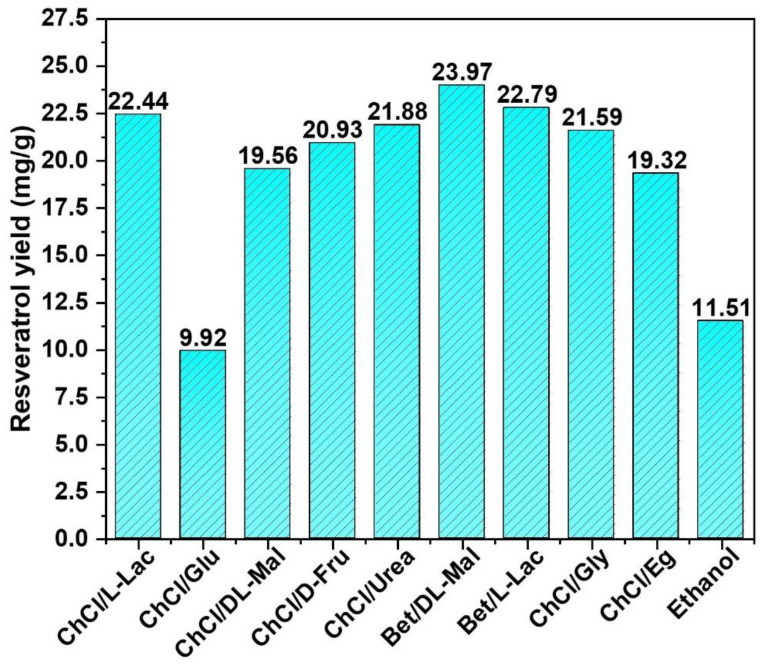
The extraction yield of resveratrol from *P. cuspidatum* using the different NADES and ethanol (60%).

As shown in Figure 2, choline chloride (ChCl), betaine (Bet), and citric acid (CA) were chosen as HBA, while L-lactic acid (L-Lac), glucose (Glu), DL-malic acid (DL-Mal), D-fructose (D-Fru), urea, glycerol (Gly), ethylene glycol (EG), and citric acid (CA) were selected as HBD. The NADES were prepared following a modified literature procedure [41,42]. Each HBD and HBA was combined at specific molar ratios (detailed in Table 1) and heated in a water bath at 80 °C with continuous stirring until a homogeneous, colorless liquid formed. After cooling to room temperature, the resulting viscous NADES were diluted with distilled water to a fixed water content of 40% (*v*/*v*). All prepared NADES solutions were stored sealed at room temperature in the dark before use.

The ultrasonic extraction parameters were set at 240 W power, 30 min duration, and 40 °C temperature. Following ultrasonic extraction, the samples were centrifuged at 10,000 rpm for 10 min [22]. The volume of the extraction liquid was recorded, and the supernatant was collected and then diluted 200−fold with anhydrous ethanol. Subsequently, the NADES was substituted with anhydrous ethanol, maintaining identical extraction conditions, to determine its yield. All subsequent measurements followed this protocol to determine the absorbance values of the extraction liquids and calculate the resveratrol yield using the standard curve formula. In total, seventeen different NADES formulations were prepared and screened as potential extraction solvents for resveratrol from *P. cuspidatum*. Among all the solvents used in the UAE, the NADES system composed of betaine/DL-malic acid had a significantly higher extraction yield of resveratrol than the other NADES and 60% ethanol due to the more hydrogen bonds between the NADES and the target compound [43]. Moreover, the acidity of betaine/DL-malic acid may have an impact on the polydatin’s glycosidic bond breaking, increasing conversion efficiency and resveratrol production [44,45]. Therefore, betaine/DL-malic acid was selected for subsequent experiments to investigate the effect of extraction conditions on resveratrol yield.

### 3.2. Effect of HBA/HBD Molar Ratio

**Figure 3:**
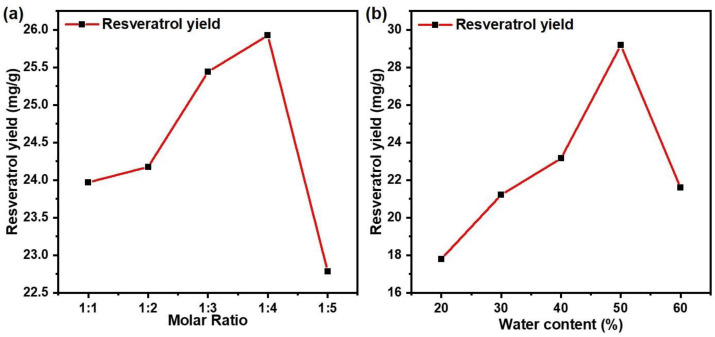
(**a**) The extraction yield of resveratrol from *P. cuspidatum* by the NADES with different ratios of HBA and HBD; (**b**) The extraction yield of resveratrol from *P. cuspidatum* by the NADES with different volumes of water to obtain the optimal water content.

As reported, the hydrogen bond interaction of the NADES was determined by the molar ratio of HBD and HBA, which affects the viscosity and surface tension of NADES. The physical properties of NADES would further affect the extraction efficiency of resveratrol [45]. Therefore, NADES based on betaine/DL−malic acid at different molar ratios were evaluated. A large number of crystals was produced when the molar ratio was from 2:1 to 5:1 in betaine and DL−malic acid. And the extraction yield rose with an increasing betaine/DL−malic acid molar ratio up to an optimum of 1:4, beyond which it decreased with any further increase (Figure 3a). Increasing the proportion of DL−malic acid in the NADES initially reduced the solvent’s viscosity and surface tension, thus facilitating diffusion and mass transfer and leading to a higher extraction yield. However, beyond an optimal point, a further increase weakened the molecular interactions between betaine and DL−malic acid, which consequently decreased the yield [46,47].

### 3.3. Water Content

It was demonstrated by Patil (2021) [48] that the high viscosity of NADES impeded the mass transfer of plant material to NADES. Accordingly, the reduction of NADES viscosity by water addition typically leads to a significant enhancement in the yield of the desired product [49]. Thus, the NADES with different volumes of water (20, 30, 40, 50, and 60%, *v*/*v*) were evaluated for the extraction of resveratrol. As shown in Figure 3b, the extraction efficiency was strongly dependent on the water content, with the maximum resveratrol yield achieved at 50% (*v*/*v*) water. At this optimal water content, the viscosity is sufficiently reduced to enhance matrix penetration without excessively diluting the hydrogen-bonding network. When the moisture content was below 50%, incomplete dissolution of the matrix compounds in the NADES occurred due to high viscosity, consequently lowering mass transfer efficiency. Conversely, the extraction yield could be diminished by excessive water, which weakens the hydrogen bond-mediated interaction between the NADES and target compound [50]. Therefore, based on the yield optimization profile, a NADES containing 50% (*v*/*v*) water was selected for all subsequent experiments.

### 3.4. Single−Factor Experiments for NADES Extraction

#### 3.4.1. Solid−Liquid Ratio

**Figure 4:**
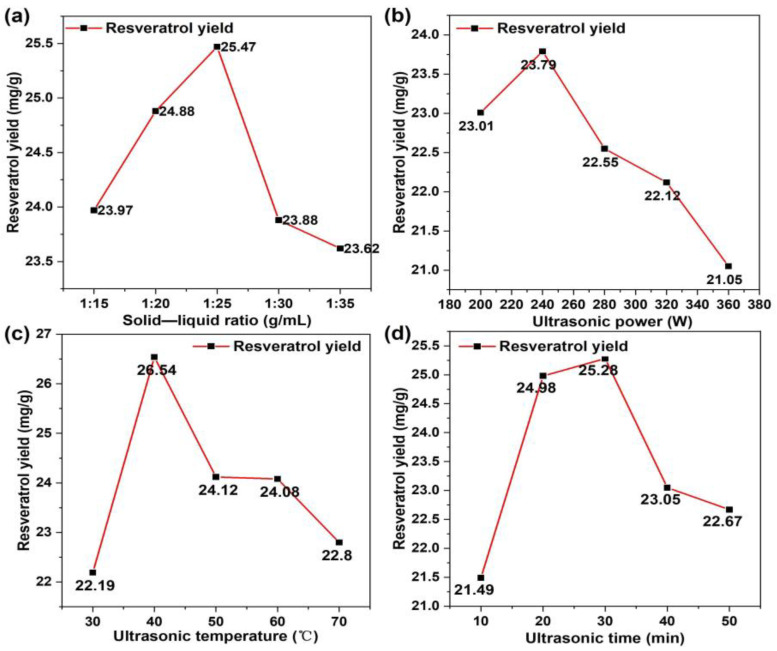
Single−factor experiment for the extraction of resveratrol. (**a**) Solid−liquid ratio; (**b**) ultrasonic power; (**c**) ultrasonic temperature; and (**d**) ultrasonic time.

The solid–liquid ratio directly influences the concentration gradient of the target compound between the plant matrix and the solvent, which serves as the primary driving force for mass transfer [51]. As shown in Figure 4a, the yield of resveratrol increased with the solvent volume and reached a maximum at a solid–liquid ratio of 1:25 (g/mL). Beyond this optimum, a further increase in the solvent volume led to a decrease in yield. This decline can be attributed to an excessive dilution of the solvent, which reduces the concentration gradient and, thus, the mass transfer efficiency [52]. Therefore, a ratio of 1:25 (g/mL) was selected for subsequent experiments to balance extraction yield with solvent economy.

#### 3.4.2. Ultrasonic Power

Ultrasonic power affects extraction by influencing cavitation intensity and cell wall disruption. As illustrated in Figure 4b, the resveratrol yield increased with ultrasonic power up to an optimum of 240 W. At this power, the cavitation effect was sufficient to enhance mass transfer and facilitate the release of intracellular compounds [53]. However, when the power exceeded 240 W, reaching 360 W, the yield decreased. This may be due to the formation of a dense cavitation zone that impedes energy transmission or, more likely, to the potential degradation of heat-sensitive compounds like resveratrol under excessively intense ultrasonic conditions [54]. Consequently, 240 W was chosen as the optimal ultrasonic power.

#### 3.4.3. Ultrasonic Temperature

Ultrasonic temperature significantly impacts both the solubility of resveratrol and its stability [55]. The yield of resveratrol initially increased with rising temperature but declined when the temperature exceeded 40 °C (Figure 4c). This reduction can be attributed to the oxidation and degradation of resveratrol at higher temperatures [56]. Considering both efficiency and compound stability, 40 °C was established as the optimal ultrasonic temperature.

#### 3.4.4. Ultrasonic Time

The influence of ultrasonic time on yield is presented in Figure 4d. The yield increased rapidly within the first 30 min as the ultrasonic energy effectively disrupted the cell structure and promoted mass transfer. Prolonging the extraction beyond 30 min led to a decrease in yield. This suggests that the extraction equilibrium was reached around this time, and further exposure to ultrasonic irradiation may have induced the degradation of resveratrol [57]. Thus, an ultrasonic time of 30 min was determined to be optimal.

### 3.5. Optimization of the Extraction Parameters by RSM

As shown in Table 2, RSM-CCD was employed to evaluate the interaction among four significant factors and to optimize the extraction conditions. These results of the orthogonal experimental design with 4 factors of 5 levels are shown in Table 3. The experimental data were fitted by a second−order polynomial model via multiple regression analysis. The resulting regression equation for the response (Y) in terms of the coded variables is:

Y = 3.20 + 0.1672A − 0.021B − 0.046C − 0.0238D − 0.0251AB − 0.0015AC − 0.0398AD − 0.0117BC + 0.013BD + 0.0171CD − 0.3487A^2^ − 0.2736B^2^ − 0.343C^2^ − 0.267D^2^

In the model, Y denotes the resveratrol yield, while A, B, C, and D represent the coded values for the solid–liquid ratio, ultrasonic power, ultrasonic temperature, and ultrasonic time, respectively.

Analysis of variance (ANOVA) confirmed the high significance of the regression model [58] (F = 56.93, *p* < 0.0001; Table 4). The non−significant Lack of Fit (*p* = 0.2090) and the high R^2^ value (0.9815 > 0.95) collectively demonstrate that the model adequately describes the experimental data and offers reliable predictions.

According to the ANOVA results (Table 4), the solid–liquid ratio (A) and ultrasonic temperature (C) were significant factors (*p* < 0.05), whereas ultrasonic power (B) and ultrasonic time (D) did not exhibit significant effects on resveratrol yield. Based on the magnitude of the F−values, the factors influencing the yield followed the order: solid–liquid ratio (A) > ultrasonic temperature (C) > ultrasonic time (D) > ultrasonic power (B). Furthermore, all quadratic terms (A^2^, B^2^, C^2^, and D^2^) were highly significant (*p* < 0.0001), suggesting pronounced nonlinear relationships in the extraction process, which is essential for interpreting the experimental outcomes [59].

**Figure 5:**
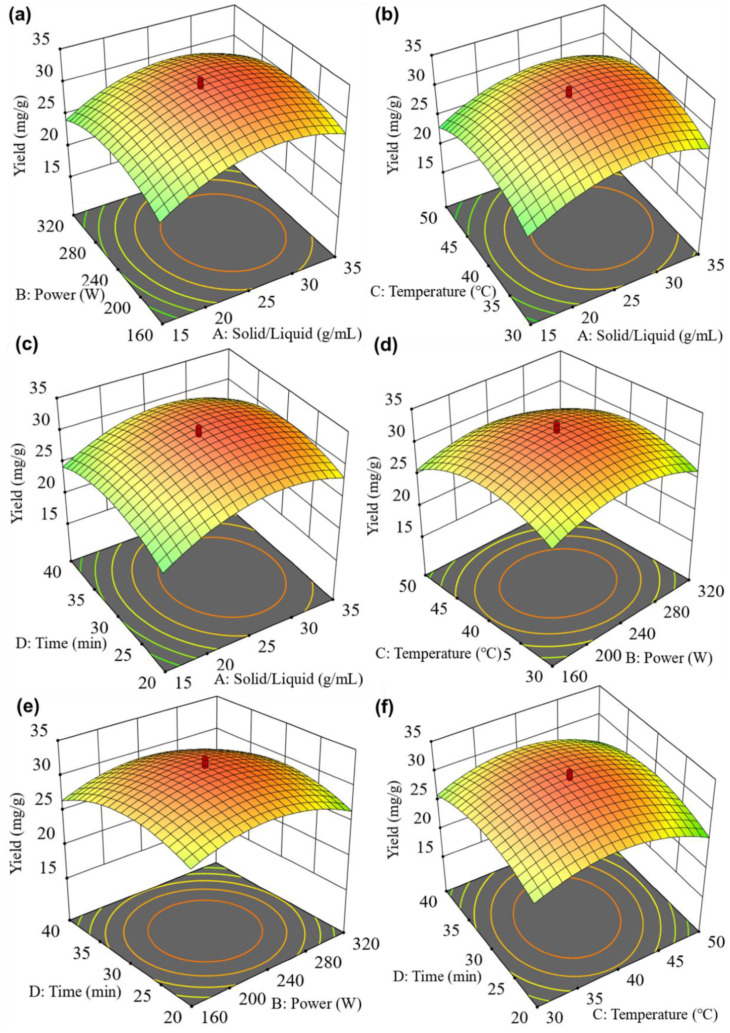
Interaction diagrams of various factors: (**a**) solid–liquid ratio and ultrasonic power; (**b**) solid–liquid ratio and ultrasonic temperature; (**c**) solid–liquid ratio and ultrasonic time; (**d**) ultrasonic power and ultrasonic temperature; (**e**) ultrasonic power and ultrasonic time; (**f**) ultrasonic temperature and ultrasonic time.

The combined effects of independent variables on resveratrol yield are visualized in the three-dimensional response surfaces (Figure 5). Each surface plot displays the extraction yield on the ordinate against two independent variables on the abscissa, illustrating their combined influence on the process. All surfaces exhibit an upward convex shape with a maximum yield located near the center, confirming the model’s validity and the presence of optimal conditions [60]. Stronger interactive effects were evident for the AB (solid–liquid ratio and ultrasonic power) and AD (solid–liquid ratio and ultrasonic time) interactions, as indicated by their steeper slopes and elliptical contours. In contrast, the AC, BC, BD, and CD interactions showed gentler slopes and more circular contours, reflecting weaker influences, which aligns with the ANOVA results.

The quadratic regression model predicted an optimal yield of 32.30 mg/g under the following conditions: solid–liquid ratio of 1:27.5 g/mL, ultrasonic power of 236.0 W, temperature of 39.3 °C, and time of 29.3 min. For practical operation, parameters were adjusted to a ratio of 1:28 g/mL, power of 240 W, temperature of 40 °C, and time of 30 min. Under these adjusted conditions, the mean experimental yield was 33.12 mg/g (n = 3). This value deviated by only 2.5% from the model prediction, demonstrating the high accuracy and practical utility of the established RSM model.

### 3.6. HPLC Analysis of Resveratrol and Other Constituents in P. cuspidatum Extract

#### 3.6.1. Determination of Sample Content

A series of resveratrol standard solutions in ethanol was prepared at concentrations of 0.1, 1, 5, 10, 20, and 50 μg/mL. The retention time and peak area of resveratrol were measured at different concentrations. A linear calibration curve was established by plotting the peak area (Y) against the concentration (X) with the regression equation Y = 57,538.74843X − 8377.04001 and a correlation coefficient (R^2^) of 0.9993, indicating excellent linearity. From the analysis of the resveratrol standard solution, its retention time was determined to be approximately 5 min. This retention time served as a reference for estimating the elution times of other components in the actual samples.

#### 3.6.2. HPLC Analysis of the Actual Extract

**Figure 6:**
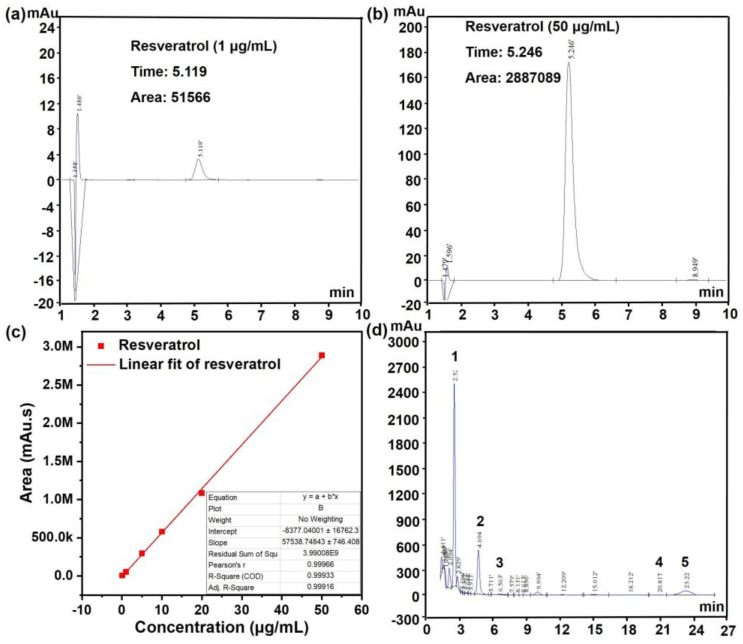
(**a**) HPLC for 1 μg/mL resveratrol in ethanol; (**b**) HPLC for 50 μg/mL resveratrol in ethanol; (**c**) standard curve for resveratrol tested by HPLC; (**d**) HPLC analysis for the actual extract from *P. cuspidatum* to assure 1: polydatin, 2: resveratrol, 3: emodin−8−O−β−D-glucoside, 4: emodin, and 5: physcion.

Polydatin elutes before resveratrol; therefore, it appears as the first major peak in the chromatogram of the *P. cuspidatum* sample. In all three sample chromatograms, a peak was consistently observed at approximately 2.7 min, which was identified as 1: polydatin [11]. Based on literature reports, emodin-8-O-β-D-glucoside elutes shortly after resveratrol. Consequently, the peak closest to the resveratrol peak was assigned as emodin-8-O-β-D-glucoside. The peak consistently observed around 7 min in the triplicate analyses was, therefore, identified as 3: emodin-8-O-β-D-glucoside [61]. Physcion (emodin monomethyl ether) is the least polar among these compounds and, therefore, has the longest retention time on the reversed-phase column [61]. A peak consistently observed around 25 min in all three chromatograms was identified as 5: physcion. Emodin has a polarity slightly greater than that of physcion, resulting in a somewhat shorter retention time. In the triplicate chromatograms, a peak was consistently observed around 21 min, with no other impurity peaks between it and the physcion peak. It was consistent with the literature [61] and, therefore, identified as 4: emodin. Therefore, we have identified the compounds in the extract as 1: polydatin, 2: resveratrol, 3: emodin-8-O-β-D-glucoside, 4: emodin, and 5: physcion. Furthermore, based on the calibration curve of resveratrol, the content of resveratrol in the extract was accurately calculated to be 2.95 mg/g (Figure 6). These values are higher than the yields of 0.25 mg/g and 2.65 mg/g reported in the literature [14,15], which clearly demonstrates the superiority of this system.

### 3.7. Antioxidant Activity of the Extract and Study Limitations

**Figure 7:**
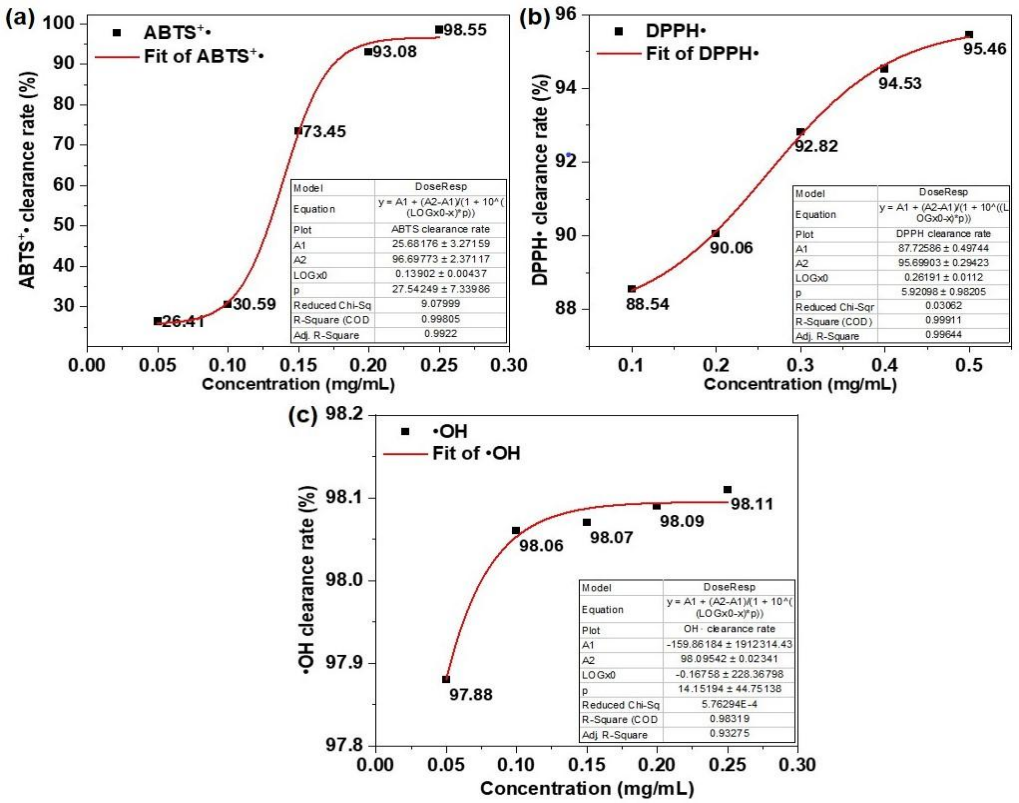
The scavenging effects of the resveratrol extract on ABTS^+^• (**a**); DPPH• (**b**); and •OH (**c**) radicals.

Resveratrol is known for its potent antioxidant activity, which primarily functions by scavenging free radicals [62]. In this study, the antioxidant capacity of the extracted resveratrol was evaluated using ABTS^+^•, DPPH•, and •OH radical scavenging assays. As shown in Figure 7, the extracted resveratrol exhibited significant and concentration-dependent radical scavenging activities across all three assays. Specifically, the ABTS^+^• scavenging rate increased from 26.41% to 98.55% as the resveratrol concentration rose from 0.05 to 0.25 mg/mL (Figure 7a). Similarly, the DPPH• scavenging activity showed a dose-dependent increase, reaching a maximum of 95.46% at 0.50 mg/mL (Figure 7b). The •OH scavenging activity also demonstrated a clear concentration dependence, achieving a rate of 98.11% at 0.25 mg/mL (Figure 7c). We have tested the antioxidant activity of only NADES and resveratrol standards as a contrast. It can be observed that a 0.1 mg/mL NADES solution exhibited antioxidant activity values of −4.12% against ABTS^+^•and −7.34% against DPPH•. This indicates that the NADES itself lacks intrinsic antioxidant properties. However, upon subsequent addition of resveratrol standard, the mixture demonstrated significant antioxidant activity against both ABTS^+^• and DPPH•. This further verifies the antioxidant capacity contributed by resveratrol within the extract.

To quantitatively compare the antioxidant potency, the half-maximal inhibitory concentration (IC_50_) values were calculated by nonlinear regression. The IC_50_ values for ABTS^+^•, DPPH•, and •OH scavenging were 0.139 ± 0.004 mg/mL, 0.026 ± 0.001 mg/mL, and 16.7 ± 2.28 × 10^−3^ mg/mL, respectively. These results confirm the strong antioxidant potential of the resveratrol obtained via the NADES-UAE method, consistent with previous reports [63,64].

## 4. Conclusions

Compared to other systems, the extraction yield of resveratrol obtained with our selected NADES system was determined to be 33.12 mg/g by UV-Vis spectroscopy and 2.95 mg/g by HPLC analysis. Both values are higher than the yields of 0.25 mg/g and 2.65 mg/g reported in the literature, which clearly demonstrates the superiority of this system. By comparing the resveratrol yields, it was found that the NADES system composed of betaine/DL−malic acid with a molar ratio of 1:4 had the highest yield. Under the optimized conditions (solid−liquid ratio of 1:28 g/mL, ultrasonic power of 240 W, ultrasonic temperature of 40 °C, and ultrasonic time of 30 min), the extraction process proved highly efficient. Furthermore, the obtained extract demonstrated potent antioxidant activity, with IC_50_ values of 0.139 mg/mL, 0.026 mg/mL, and 0.0167 mg/mL against ABTS^+^•, DPPH•, and •OH radicals, respectively.

This study establishes NADES−UAE as an effective, straightforward, and environmentally benign method. The technique provides a sustainable and high-yield strategy for obtaining resveratrol from *P. cuspidatum*, showing considerable promise for the production of natural functional food ingredients and nutraceuticals.

## Figures and Tables

**Table 1:** Various types of synthesized NADES.

NO.	HBA	HBD	Molar Ratio	Appearance at Room Temperature
NADES-1	ChCl	L-Lac	1:1	Crystallized
NADES-2	ChCl	L-Lac	1:2	Clear liquid
NADES-3	ChCl	Glu	1:1	Solid–liquid mixture
NADES-4	ChCl	Glu	1:2	Clear liquid
NADES-5	ChCl	DL-Mal	1:1	Clear liquid
NADES-6	ChCl	D-Fru	1:1	Clear liquid
NADES-7	ChCl	Urea	1:1	Crystallized
NADES-8	ChCl	Urea	1:2	Clear liquid
NADES-9	Bet	DL-Mal	1:1	Clear liquid
NADES-10	Bet	L-Lac	1:1	Clear liquid
NADES-11	ChCl	Gly	1:1	Crystallized
NADES-12	ChCl	Gly	1:2	Clear liquid
NADES-13	ChCl	EG	1:1	Crystallized
NADES-14	ChCl	EG	1:2	Clear liquid
NADES-15	ChCl	CA	1:1	Solid–liquid mixture
NADES-16	ChCl	CA	1:2	Solid–liquid mixture
NADES-17	CA	D-Fru	1:1	Solid–liquid mixture

**Table 2:** Factors and levels studied using Central Composite Design (CCD).

Factors	Coded Symbols	Levels				
		−2	−1	0	1	2
Solid–liquid ratio (g/mL)	A	1:5	1:15	1:25	1:35	1:45
Ultrasonic power (W)	B	80	160	240	320	400
Extraction temperature (°C)	C	20	30	40	50	60
Extraction time (min)	D	10	20	30	40	50

**Table 3:** Experimental design and results of Central Composite Design (CCD).

Std	A (g/mL)	B (W)	C (°C)	D (min)	Yields (mg/g)
1	15	160	30	20	1.779
2	35	160	30	20	2.397
3	15	320	30	20	1.938
4	35	320	30	20	2.059
5	15	160	50	20	1.806
6	35	160	50	20	2.232
7	15	320	50	20	1.605
8	35	320	50	20	2.149
9	15	160	30	40	1.816
10	35	160	30	40	2.107
11	15	320	30	40	1.733
12	35	320	30	40	2.105
13	15	160	50	40	1.827
14	35	160	50	40	2.083
15	15	320	50	40	1.794
16	35	320	50	40	1.948
17	5	240	40	30	1.524
18	45	240	40	30	2.139
19	25	80	40	30	2.079
20	25	400	40	30	2.185
21	25	240	20	30	2.008
22	25	240	60	30	1.701
23	25	240	40	10	2.163
24	25	240	40	50	2.154
25	25	240	40	30	3.212
26	25	240	40	30	3.309
27	25	240	40	30	3.193
28	25	240	40	30	3.186
29	25	240	40	30	3.078
30	25	240	40	30	3.249

**Table 4:** ANOVA statistics of the quadratic model for resveratrol extraction ability by NADES.

Source	Sum of Squares	Degree of Freedom	Degree of Freedom	F-Value	*p*-Value	Significant
**Model**	826.35	14	59.03	56.93	<0.0001	significant
A—solid–liquid	67.06	1	67.06	64.68	<0.0001	**
B—Power	1.06	1	1.06	1.02	0.3287	
C—Temperature	5.09	1	5.09	4.90	0.0427	*
D—Time	1.35	1	1.35	1.31	0.2709	
AB	1.01	1	1.01	0.9731	0.3395	
AC	0.0038	1	0.0038	0.0037	0.9526	
AD	2.53	1	2.53	2.44	0.1391	
BC	0.2198	1	0.2198	0.2120	0.6518	
BD	0.2697	1	0.2697	0.2601	0.6175	
CD	0.4679	1	0.4679	0.4513	0.5119	
A^2^	333.57	1	333.57	321.70	<0.0001	
B^2^	205.32	1	205.32	198.02	<0.0001	
C^2^	322.66	1	322.66	311.19	<0.0001	
D^2^	195.50	1	195.50	188.55	<0.0001	
Residual	15.55	15	1.04			
Lack of Fit	12.60	10	1.26	2.13	0.2090	not significant
Pure Error	2.96	5	0.5913			
Cor Total	841.91	29				
Std. Dev.	1.02		R^2^		0.9815	
Mean	22.19		Adjusted R^2^		0.9643	
C.V. %	4.59		Predicted R^2^		0.9088	
			Adeq Precision		24.0164	

**: Very significant; *: Significant.

## Data Availability

The original contributions presented in this study are included in the article. Further inquiries can be directed to the corresponding authors.

## Abbreviations

The following abbreviations are used in this manuscript:

NADES	natural deep eutectic solvent
HSCCC	high-speed counter-current chromatography
HBA	hydrogen bond acceptor
HBD	hydrogen bond donor
CCD	central composite design
RSM	response surface methodology
DPPH	2,2-diphenyl-1-picrylhydrazyl
ABTS	2,2′-azino-bis(3-ethylbenzothiazoline-6- sulfonic acid
UAE	ultrasound-assisted extraction
